# Material flow composition dataset: RGB-camera-based single-particle images and particle mass data of shredded WEEE

**DOI:** 10.1016/j.dib.2025.112413

**Published:** 2025-12-22

**Authors:** Malte Vogelgesang, Alice do Carmo Precci Lopes, Emanuel Ionescu, Liselotte Schebek

**Affiliations:** a Fraunhofer Research Institution for Materials Recycling and Resource Strategies IWKS, Brentanostr. 2a, Alzenau D-63755, Germany; b Institute IWAR, Technical University Darmstadt, Franziska-Braun-Str. 7, Darmstadt D-64287, Germany

**Keywords:** Waste electrical and electronic equipment, E-waste, Material flow characterization, Particle mass prediction, Sensor-based sorting, Machine learning, Computer vision, Recycling

## Abstract

Mass-based material flow compositions (MFCOs) are among the most relevant indicators for the quality of sorting processes in waste management. They provide the basis for the assessment and improvement of the purity and yield of material streams. While MFCOs are currently primarily determined through labor-intensive manual sorting analyses, recent research has explored sensor-based material flow characterization (SBMC). Near-infrared spectroscopy (NIR) is effective for plastic waste but cannot reliably distinguish many material types in shredded waste electrical and electronic equipment (WEEE). Therefore, this article aims to provide data for research on SBMC with more versatile and often already implemented RGB cameras in sorting plants. The generated data contain shredded WEEE, which was recorded on a sensor-based sorting machine on an industrial scale. The samples were taken from an industrial WEEE recycling facility in Germany and manually presorted into four main material types. From these sorted materials, different subsamples were created according to each dataset’s objective. The three datasets consist of cropped RGB line-scan camera images and mass measurements. Dataset 1 contains labeled single-particle images for the training of image classification, object detection, and segmentation networks and is organized by material type (ferrous, non-ferrous, printed circuit board, and plastic) and by particle size (12.5 mm – 25 mm and 25 mm – 50 mm). Dataset 2 follows the same structure and provides single-particle images together with per-object masses for the training of regression models for particle mass prediction. Dataset 3 provides single-particle images from three mixed samples with ground-truth compositions to validate models trained on Datasets 1 and 2. To serve as an additional validation dataset, Dataset 1 also includes the mass of each material fraction. These datasets enable the development and evaluation of models for the identification of material types in shredded WEEE, for particle mass prediction, and sensor-based material flow characterization, supporting improved process monitoring, quality control, and optimization of sorting processes.

Specifications Table

**Table:** 

Subject	Engineering & Materials science
Specific subject area	Sensor-based material flow characterization in mechanical recycling processes
Type of data	Table (raw, processed), Images (raw, cropped)
Data collection	The datasets were generated using an industrial-scale sensor-based sorting machine (Sesotec Varisort Compact [Schoenberg, Germany]). Two parallel RGB line-scan cameras capture particles mid-flight after leaving a conveyor belt. Raw camera data were acquired in Python, processed to detect objects, and cropped accordingly. Particle masses were measured with a digital precision balance (KERN & SOHN EWJ 3000–2 [Balingen, Germany]). Masses of sample fractions were measured with a digital platform scale (KERN & SOHN DS 100K0.5 [Balingen, Germany]). To create the samples for Dataset 3, a digital precision balance was used (PCE-BSH 10000, PCE Instruments [Meschede, Germany]).
Data source location	Fraunhofer Research Institution for Materials Recycling and Resource Strategies IWKS, Brentanostr. 2a, Alzenau d-63755, Germany (50°05′25″N 9°03′04″E)
Data accessibility	Repository name: TUdatalib Data identification number: 10.48328/tudatalib-1743 Direct URL to mass data and cropped image data: https://doi.org/10.48328/tudatalib-1743 [2] Direct URL to raw image data: https://tudatalib.ulb.tu-darmstadt.de/handle/tudatalib/4842 [3]
Related research article	M. Vogelgesang, V. Kaczmarek, A.d.C.P. Lopes, C. Li, E. Ionescu, L. Schebek, Automated material flow characterization of WEEE in sorting plants using deep learning and regression models on RGB data, WASTE MANAGEMENT 204 (2025) 114904. https://doi.org/10.1016/j.wasman.2025.114904. [1]

## 1 Value of the Data

• The datasets [2] provide single-particle RGB line-scan images with per-particle and fraction-level masses from shredded waste electrical and electronic equipment (WEEE) sampled at the WEEE treatment plant of Electrocycling GmbH. The data were acquired on an industrial-scale Sesotec Varisort Compact sensor-based sorting machine and enable the reproducible development, benchmarking, and validation of sensor-based material flow characterization (SBMC) of WEEE compositions with equipment already common in sorting plants [1].
• A key contribution is enabling RGB-only workflows for particle-based material type identification and mass prediction. Compared with manual sorting analyses, the datasets provide a scalable, reproducible path to inline, mass-based characterization of WEEE, supporting closed-loop process control, the calibration of digital twins of sorting stages, and transfer learning to related streams (e.g., mixed metals, shredder residue).
• Researchers (e.g., machine learning and data scientists, recycling/process/environmental engineers) can apply these data in computer vision, machine learning (classification, detection, segmentation, regression), process control, and material flow analysis. The datasets directly support the development of material type identification (DS1), particle mass prediction (DS2), and the validation of material flow characterization with mixed samples of known compositions (DS3) [1].
• WEEE recyclers and plant operators can use the data to prototype and calibrate inline monitoring of purity, yield, and recovery for quality assurance and adaptive process control. Suppliers of sensor and sorting technology can develop and benchmark RGB-based detection and monitoring of WEEE streams, introducing the application of RGB sensors for a wide variety of waste streams. Robotics and AI companies can improve recognition systems for WEEE and related material mixtures and develop mass-based picking strategies.

## 2 Background

The data [2,3] were generated to support the development of a sensor-based material flow characterization (SBMC) of waste electrical and electronic equipment (WEEE) at sorting plants [1]. The SBMC aims at automatically analyzing material flow compositions of input and output streams at sorting stages, providing a basis for modeling and optimizing sorting plants.

The datasets provided the foundation for this development in the related research article [1]. First, the material type of each particle was identified by using three different methods: image classification, object detection, and instance segmentation (Dataset 1). From the particles’ material type and geometric properties, their masses were predicted via regression models (Dataset 2). The masses for all particles of a certain material type were then aggregated to calculate the material flow composition. The results of the models were then validated using data from mixed samples with known compositions (Dataset 3).

With publishing the data, other researchers can replicate the work and advance the area of SBMC for WEEE with their own developments.

## 3 Data Description

The datasets [2] contain cropped image files of particles from shredded waste electrical and electronic equipment. Depending on the dataset, information about the masses of materials and particle size fractions, or individual particles, was included.

**Fig. 1:**
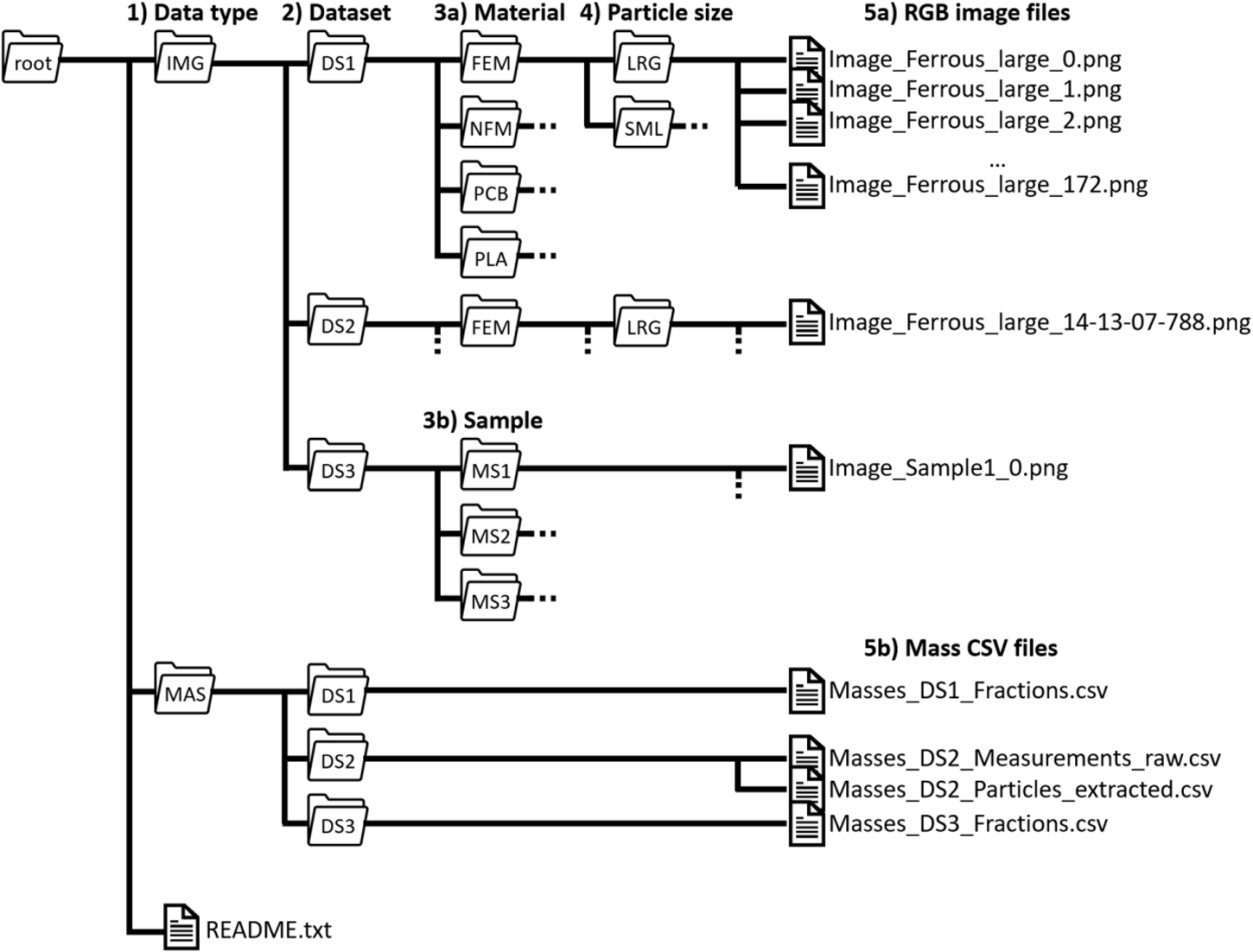
Structure of folders containing images, mass data for three datasets, and README file (DS = dataset; FEM = ferrous metal; IMG = image; LRG = large particles; MAS = mass; MS = mixed sample; NFM = non-ferrous metal; PCB = printed circuit board; PLA = plastic; SML = small particles).

The folder structure for the three datasets is presented in Fig. 1. The top-level directory contains two folders: images (**IMG**) and mass data (**MAS**). Each folder is divided into Dataset 1 (**DS1**), Dataset 2 (**DS2**), and Dataset 3 (**DS3**). On top level of the directory, a README.txt file describes the structure, naming convention, and contents of the datasets.

**Fig. 2:**
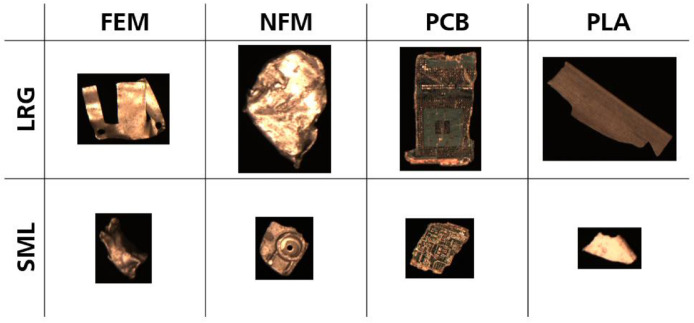
Example of cropped RGB images from Dataset 1, randomly selected from each combination of material type and particle size range (FEM = ferrous metal; NFM = non-ferrous metal; PCB = printed circuit board; PLA = plastic; LRG = 25 mm – 50 mm; SML = 12.5 mm – 25 mm).

In **Datasets 1 and 2**, images are further separated by material type: ferrous metals (**FEM**), non-ferrous metals (**NFM**), printed circuit boards (**PCB**), and plastics (**PLA**). Reflecting particle sizes from a sieving analysis, each material folder contains two image subfolders: large particles (**LRG**; 25 mm – 50 mm) and small particles (**SML**; 12.5 mm – 25 mm). Based on the material type and particle size, the images are named in the format “Image_[*MaterialType*]_[*ParticleSize*]_[*counter*].png”. In **Dataset 1**, counters are consecutive integers starting at 0. In **Dataset 2**, filenames include the image acquisition timestamp (HH-mm-ss-SSS) instead of a counter to enable matching with particle weight measurements. **Dataset 3** does not separate images by material. Instead, it is divided into folders for three mixed samples (**MS1** – **MS3**), each containing all images of the mixed material types. These images are named “Image_Sample[*number*]_[*counter*].png”. Examples of the images are shown in Fig. 2.

In the **MAS** folder, each dataset subfolder contains a CSV file with the corresponding masses. For **Datasets 1**, the files are named “Masses_DS[*number*]_Fractions.csv”. After the header, each of eight rows corresponds to a combination of material type and particle size range. The three columns are: material type (FEM, NFM, PCB, PLA), particle size range (LRG, SML), and the measured mass, rounded to whole grams. **Dataset 2** includes two mass files. The first, “Masses_DS2_Measurements_raw.csv,” contains raw particle-weight measurements: after the header, the two columns show the timestamp (HH-mm-ss-SSS) and the mass measured on the balance in grams with two decimal places. The second file, named “Masses_DS2_Particles_extracted.csv”, directly shows masses of particles that were extracted by correlating single-particle image timestamps and mass increases in the measurements: after the header, each row lists the image timestamp and the calculated mass in grams. **Dataset 3** follows the file naming convention of Dataset 1. Each of the twelve rows after the header contain a combination of mixed sample and material type. The three columns are: sample (1, 2, 3), material type (see DS1), and measured mass, rounded to whole grams.

In total, the folders and files contain the following number of images and mass entries at a size of 262 MB (Table 1).

**Table 1:** Number of PNG image files per IMG subfolder.

Data type	Dataset	Subfolder	FEM	NFM	PCB	PLA	Mixed
**IMG**							
	DS1						
		LRG	173	382	324	234	
		SML	3126	3068	538	1253	
	DS2						
		LRG	178	268	295	239	
		SML	343	267	286	367	
	DS3						
		MS1					559
		MS2					1267
		MS3					963
							

Data type	Dataset	Subfolder	Entries	Description			

**MAS**							
	DS1		8	4 material types in 2 particle size ranges			
	DS2						
		raw	82068	Time series of weight measurements			
		extracted	1976	Particle masses			
	DS3		12	4 material types in 3 samples			

Number of entries in MAS CSV files (DS = dataset; FEM = ferrous metal; IMG = image; MAS = mass; MS = mixed sample; NFM = non-ferrous metal; PCB = printed circuit board; PLA = plastic; LRG = 25 mm – 50 mm; SML = 12.5 mm – 25 mm).

The **raw image files** [3] follow the IMG folder structure described for [2] and are split into separate zip archives due to their size. DS1 (19.8 GB) contains material type folders (FEM, NFM, PCB, PLA), each with subfolders for large (LRG) and small (SML) particles. DS2 is split into separate archives by material type (DS2_FEM, 30.2 GB; DS2_NFM, 38.5 GB; DS2_PCB, 39.1 GB; DS2_PLA, 31.3 GB), each with LRG and SML subfolders. DS3 (6.3 GB) is organized by mixed samples (MS1 – MS3). At the lowest level file names are RAWDATA_[*counter*]_Camera_[*number*].png for DS1 and DS3, and RAWDATA_[*timestamp*]_Camera_[*number*].png for DS2 to enable matching with particle weight measurements. The camera number (0 or 1) indicates which camera captured the image, with 0 = left and 1 = right, as viewed in the direction of transport.

## 4 Experimental Design, Materials and Methods

The experimental design, materials, and methods described here were developed and applied in the related research article [1]. The following methods are summarized from that research article, with adaptations focused on dataset construction.

### 4.1 Sampling and sample preparation

This section describes how the source materials were obtained and prepared for data acquisition. It covers the sampling campaign at a WEEE facility, sieving into target size ranges, manual sorting by material type with inclusion/exclusion criteria, and the creation of the inputs for the three datasets.

#### 4.1.1 Sampling campaign

In November 2023, a one-day sampling campaign was carried out at the WEEE treatment facility of Electrocycling GmbH (Goslar, Germany).

The facility performs manual presorting to remove items containing glass, wood, batteries, and other contaminants. The remaining WEEE undergoes the following mechanical processes: items are crushed in a pre-shredder (i), followed by magnetic separation for large ferrous-metal fragments (ii), leaving a high-volume mixed material stream (iii) that runs through air classification to extract large plastic pieces (iv). After screening, the remaining particles are further shredded according to size. Following additional screening steps, magnetic and eddy-current separation are applied to the different particle size ranges and at different intensities to recover ferrous metals (v, vi), retaining an intermediate stream rich in non-ferrous metals and plastics (vii). From this stream, non-ferrous metals are recovered through eddy-current separation (viii-x), some of which are further refined using wet and dry density separation processes, leaving plastic-rich residue for disposal (xi-xiii).

**Fig. 3:**
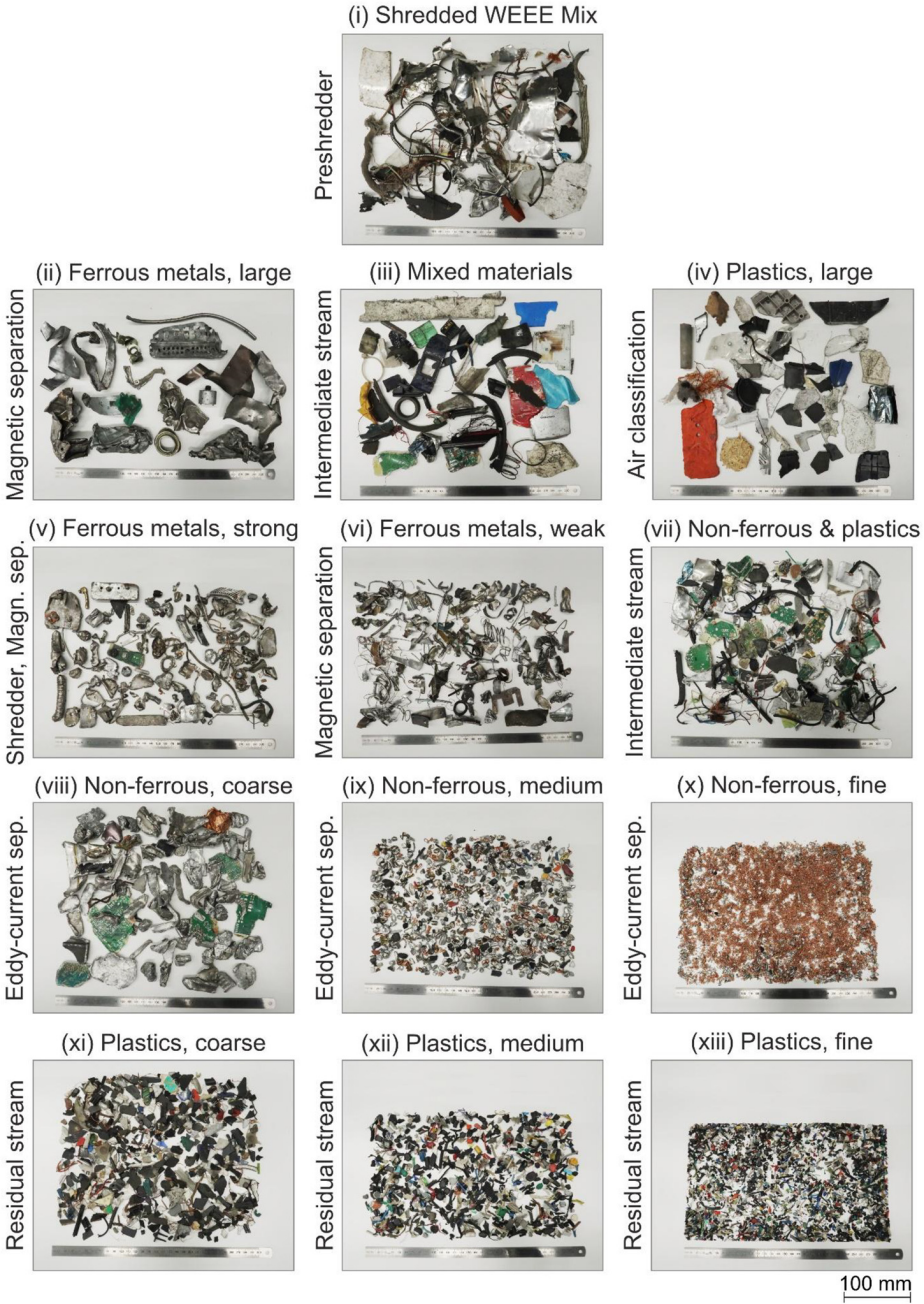
Sampled fractions from WEEE treatment facility (exemplary pictures).

Samples were collected from thirteen intermediate and output fractions from the mechanical processing line to capture most material types at the sorting plant (Fig. 3). Depending on particle size and relevance for imaging tasks, sample masses per fraction ranged from 2.6 kg to 24.9 kg. The description of each sampling point follows.

One sample was collected directly after pre-shredding (i). Additional samples were taken after the automated presorting of large ferrous metal particles (ii), from the remaining mixed stream (iii), and from the large recovered plastic fragments (iv). As the pre-shredder was idle during the sampling window, these fractions were sampled from stockpiles. From these stockpiles, composite samples were collected with a shovel from multiple locations across the surface and within the pile. Nine additional samples were collected downstream of the second comminution stage, at the outputs of the magnetic and eddy-current separation steps. Sampling was performed from the full, continuously falling waste stream in accordance with LAGA PN 98 [4]. The samples include two ferrous fractions (strongly (v) and weakly magnetic (vi)), the intermediate stream rich in plastics and non-ferrous metals stream (vii), and three non-ferrous fractions (coarse (viii), medium (ix), and fine cable shredder material (x)). The three final samples are fine-to-very-fine residues largely composed of plastics (xi-xiii).

#### 4.1.2 Material selection

At the Fraunhofer Research Institution for Materials Recycling and Resource Strategies IWKS (Alzenau, Germany), the samples were manually sieved in batches using stacked woven wire test sieves (RETSCH [Haan, Germany]; 305 mm frame diameter). Each batch was sieved for 30 s using a horizontal circular motion. All samples were screened into four particle size ranges: < 12.5 mm, 12.5 mm – 25 mm, 25 mm – 50 mm, and > 50 mm. Subsequent steps focused on the ranges of 12.5 mm – 25 mm and 25 mm – 50 mm, as these represent the majority of marketed materials and correspond to the main fractions sorted during the mechanical processing prior to wet and dry density separation.

**Fig. 4:**
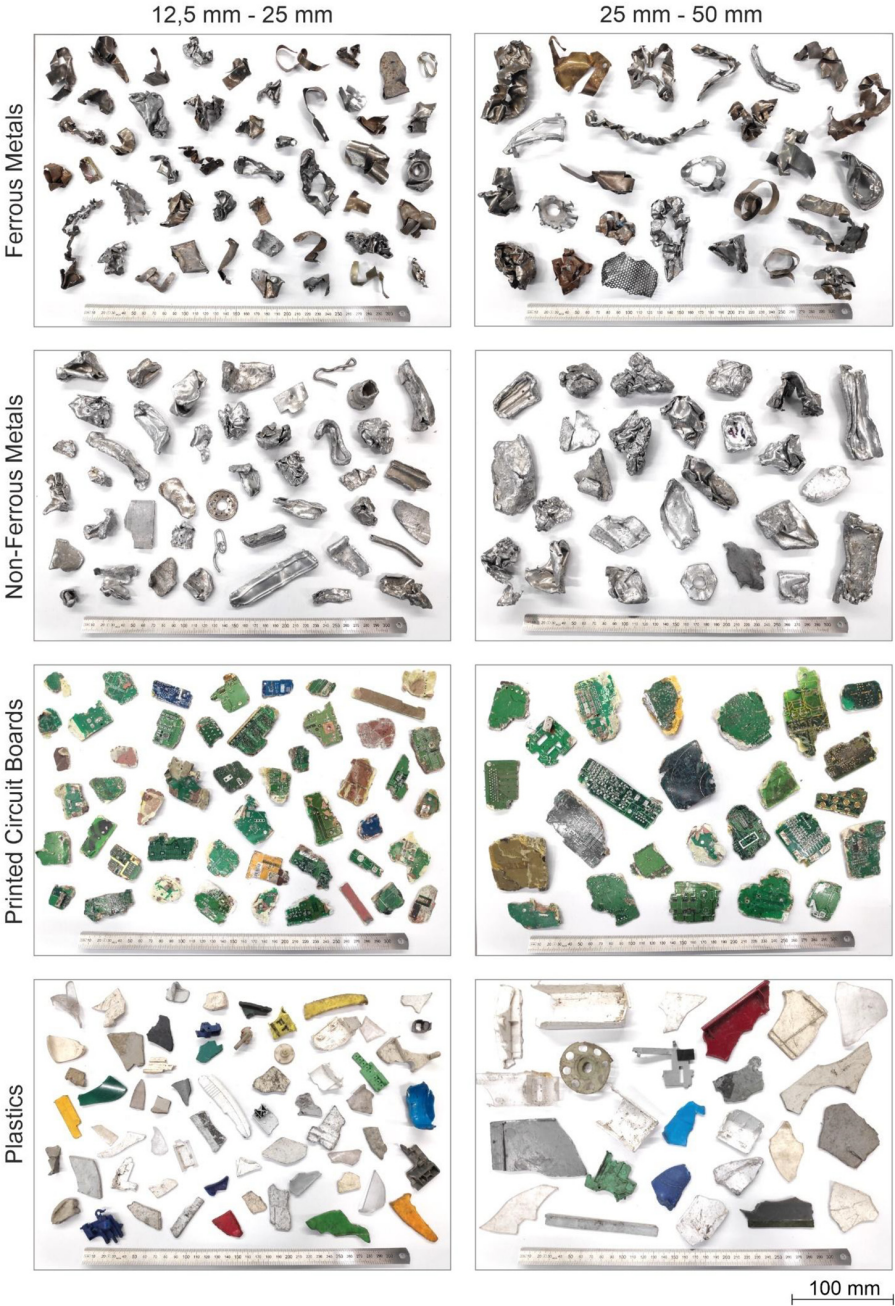
Manually sorted fractions from WEEE sample (exemplary pictures) (Reproduced from [1]. © 2025 The Authors. Licensed under CC BY 4.0).

The sieved fractions were then manually sorted by material type into ferrous metals, non-ferrous metals, PCBs, plastics, mixed-material objects, and other materials. Mixed-material objects contained more than one material, e.g., ferrous screws embedded in ball-shaped non-ferrous particles resulting from deformation in the hammer mill, or plastic and metal pieces still connected by screws and not fully separated during comminution. Manual sorting was performed to establish ground-truth for the development of material type detection models, thus removing mixed-material objects. To improve detectability in RGB images on a black belt, black objects were removed (mainly plastics and a small share of circuit boards). To reduce visual confusion with plastics, colored metal pieces were also excluded. A small subset of solid ferrous pieces was removed, because they were visually indistinguishable from non-ferrous metals. As in the related research article [1], the retained proportions were approximately 80 % of ferrous, 65 % non-ferrous, 93 % PCBs, and 64 % plastic, yielding about 75 % remaining mass across the two target size ranges for all classes. Single-material particles were then combined by particle size range and material type across the originating fractions (Fig. 4).

#### 4.1.3 Dataset creation

The manually sorted fractions were used to create three datasets (Table 2): **Dataset 1** for material type identification (MTI), **Dataset 2** for particle mass prediction (PMP), and **Dataset 3** for the prediction and validation of material flow compositions (MFCO).

**Table 2:** Dataset sample compositions in mass (kg) or particle count (n).

			FEM	NFM	PCB	PLA	Sum
**DS1**							
		LRG	1.823 kg	4.093 kg	1.774 kg	1.079 kg	8.769 kg
		SML	14.161 kg	11.407 kg	1.098 kg	1.396 kg	28.062 kg
		Sum	15.984 kg	15.500 kg	2.872 kg	2.475 kg	36.831 kg
**DS2**							
		LRG	*n* = 178	*n* = 268	*n* = 295	*n* = 239	*n* = 980
		SML	*n* = 343	*n* = 267	*n* = 286	*n* = 367	*n* = 1263
		Sum	*n* = 521	*n* = 535	*n* = 581	*n* = 606	*n* = 2243
**DS3**							
	MS1						
		LRG	0.300 kg	0.302 kg	0.201 kg	0.202 kg	1.004 kg
		SML	0.300 kg	0.299 kg	0.200 kg	0.200 kg	0.999 kg
		Sum	0.599 kg	0.601 kg	0.401 kg	0.402 kg	2.003 kg
	MS2						
		LRG	0.999 kg	0.199 kg	0.501 kg	0.501 kg	2.005 kg
		SML	0.802 kg	0.400 kg	0.101 kg	0.699 kg	2.001 kg
		Sum	1.802 kg	0.599 kg	0.406 kg	1.200 kg	4.006 kg
	MS3						
		LRG	0.206 kg	1.002 kg	0.492 kg	0.299 kg	1.999 kg
		SML	0.400 kg	0.803 kg	0.700 kg	0.101 kg	2.003 kg
		Sum	0.606 kg	1.804 kg	1.192 kg	0.400 kg	4.002 kg

(DS = dataset; FEM = ferrous metal; MS = mixed sample; NFM = non-ferrous metal; PCB = printed circuit board; PLA = plastic; LRG = 25 mm – 50 mm; SML = 12.5 mm – 25 mm) (Adapted from [1]).

**Dataset 1 (MTI):** All particles in each material category and particle size range of the presorted fractions were recorded for the training of models for the optical identification of material types. The recording was performed on a sensor-based sorting machine with the sorting program disabled (see Section 3.2.1). The samples were fed to the vibratory feeder in bulk and the recorded images were cropped to individual particles as described in Section 3.2.2. The mass of each fraction was measured with a platform scale (KERN & SOHN DS 100K0.5 [Balingen, Germany]) and rounded to the next integer in grams.

**Dataset 2 (PMP):** This dataset provides paired images and masses of individual particles for the training of particle mass prediction models using material type and geometric features. To keep acquisition time practical and balance counts across groups, recording was limited to 20 min per combination of material type and size, or until that combination was exhausted. To preserve representativeness, a ripple divider was used to produce portions of approximately 100 particles, which were fed to the sensor-based sorting machine sequentially. The particles were manually placed, one by one, on the chute feeding the machine (see Section 3.2.3). Single-particle images were extracted using the same routine as for the other datasets (Section 3.2.2) and saved with acquisition timestamps in their filenames. Per-particle masses were obtained using the synchronization method described in Section 3.2.4. In total, 1976 of 2243 single-particle images (88 %) were matched to corresponding masses. The resulting per-particle masses and the raw time series are provided. After data acquisition, subsamples were returned to their parent fractions, to be used for Dataset 3.

**Dataset 3 (MFCO):** Mixed samples of predefined compositions were created to validate MFCO predictions from models developed on Datasets 1 and 2. A ripple divider was used to generate subfractions small enough to be combined at the intended ratios. Mixed Sample 1 and also the sum of all three Mixed Samples consist of 30 % ferrous metals, 30 % non-ferrous metals, 20 % plastics, and 20 % PCBs, approximating a typical WEEE composition of 50 % metals, 27 % plastics, and 23 % other components (including PCBs) [5]. Mixed Samples 2 and 3 intentionally deviate from this baseline to test robustness to stream fluctuations. The different materials were mixed thoroughly by hand before recording the images and extracting the objects identically to Dataset 1. Component masses were weighed on a precision balance with 0.2 g resolution (PCE-BSH 10,000, PCE Instruments [Meschede, Germany]) [1].

### 4.2 Setup and data acquisition

This section describes the setup used for imaging and weighing and presents the data acquisition workflow. It includes the image preprocessing and object extraction pipeline and the method used to synchronize image timestamps with weight measurements.

#### 4.2.1 Imaging setup

**Fig. 5:**
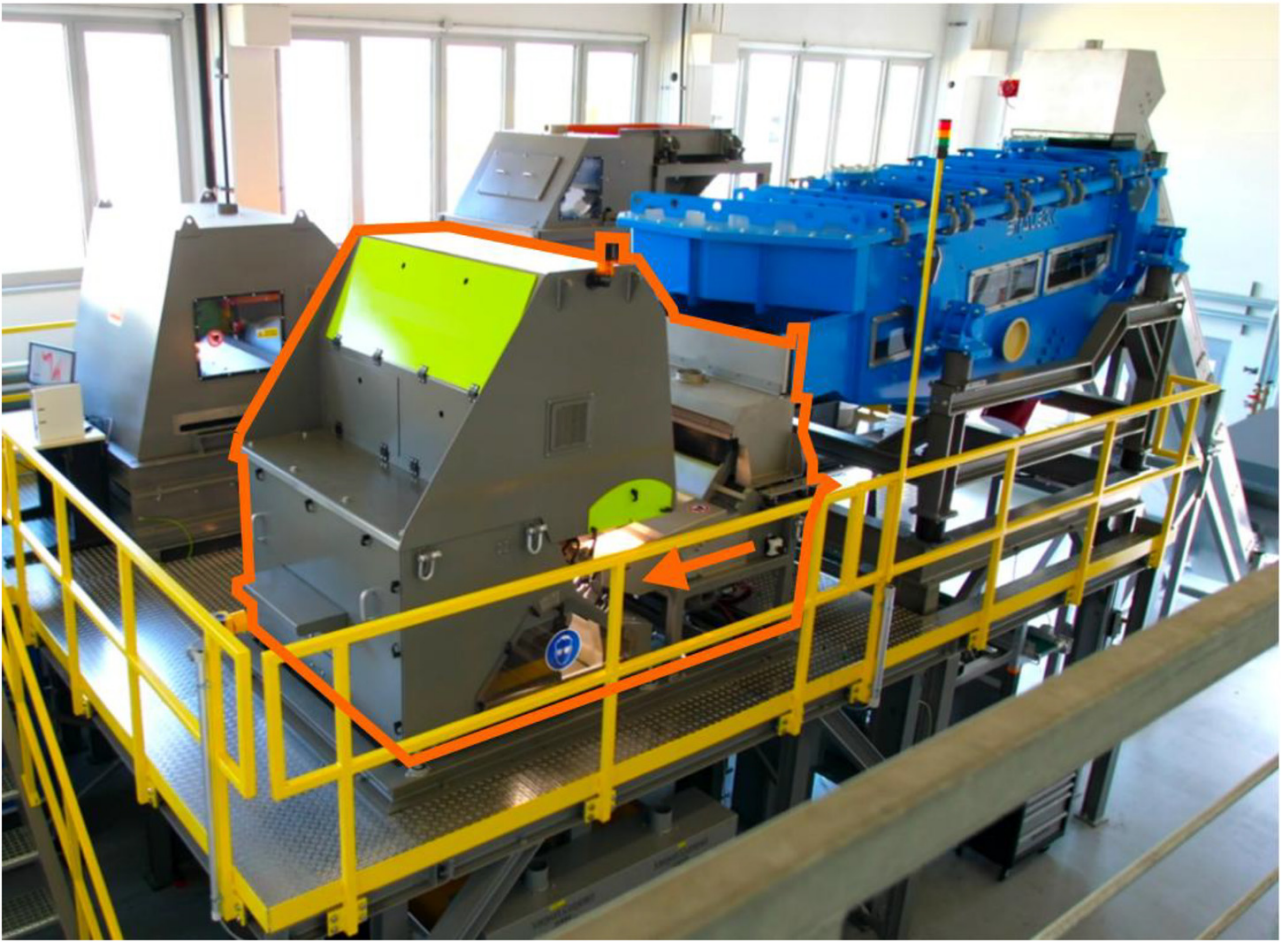
Modular sorting plant at Fraunhofer IWKS (Alzenau, Germany); the multi-sensor sorting system is highlighted in orange (Reproduced from [1]. © 2025 The Authors. Licensed under CC BY 4.0).

Images were acquired on an industrial-scale sensor-based sorting machine (Sesotec Varisort Compact [Schoenberg, Germany]), which is part of the modular sorting plant at Fraunhofer IWKS Alzenau, Germany (Fig. 5). The system is equipped with two parallel RGB line-scan cameras (1365×1 pixels) operated under LED illumination, covering the full 1024 mm belt width. Due to the parallax between the two cameras, optical distortions can appear in the narrow overlapping area, especially for 3D objects. To increase data quality during image acquisition, a mechanical divider was placed at the center of the vibratory feeder’s chute, keeping about 5 % of the belt’s width free of particles. Additional sensors (inductive electromagnetic and hyperspectral near-infrared) were present but not used in this study [6]. The compressed-air ejection was disabled, as the objective was data acquisition only.

**Fig. 6:**
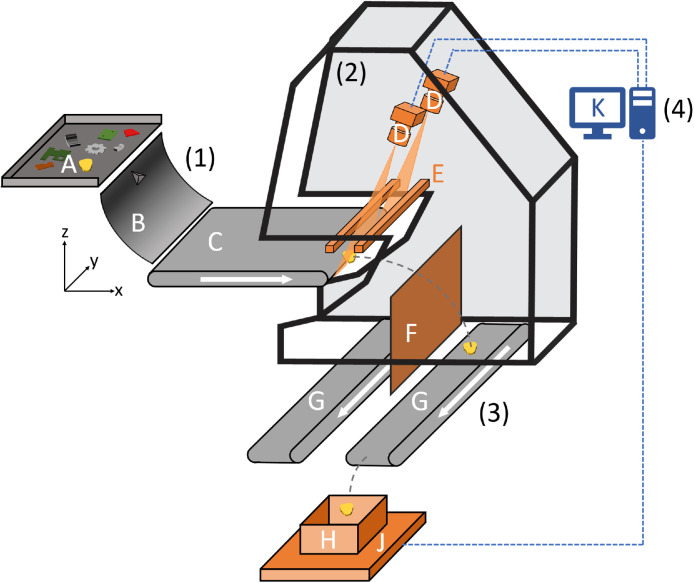
Schematic drawing of the setup for data acquisition; Components: (1) Feeding, (2) RGB image acquisition, (3) weighing, (4) data recording and processing; A. vibratory feeder with particles, B. chute with divider, C. accelerating conveyor, D. RGB line-scan cameras, E. LED illumination, F. separating vertex, G. output conveyors, H. collecting container, J. digital balance, K. computer hardware, L. halogen illumination for NIR sensor, M. inductive sensor, N. NIR sensor, O. pneumatic ejection system (Adapted from [1]; redrawn to increase resolution. © 2025 The Authors. CC BY 4.0).

The samples were manually fed onto the vibratory feeder, dispersed, and accelerated via the chute onto the conveyor running at 1.8 m/s (Fig. 6). Imaging was performed at the end of the belt as particles left the conveyor and passed through the cameras’ field of view. The RGB line-scan cameras were operated with the manufacturer’s factory presets regarding white/black balance and shading correction. The scan rate of the cameras was calibrated to produce square pixels, resulting in a spatial resolution of 0.38 mm/pixel. Illumination was provided by the integrated LED lighting at 40 % intensity, based on preliminary tests showing the best results for the investigated materials at the chosen scan rate. See [1] for additional details.

#### 4.2.2 Image preprocessing and object extraction

During acquisition, the RGB line-scan data from each camera were segmented into frames of 500 lines [3]. Frames were processed in two steps: first, an overlapping **sliding window technique** to avoid split or duplicated objects in the images, then **particle extraction and image processing** to remove empty images and background through cropping. Image processing was implemented in Python using OpenCV [7] and scikit-image [8].

**Fig. 7:**
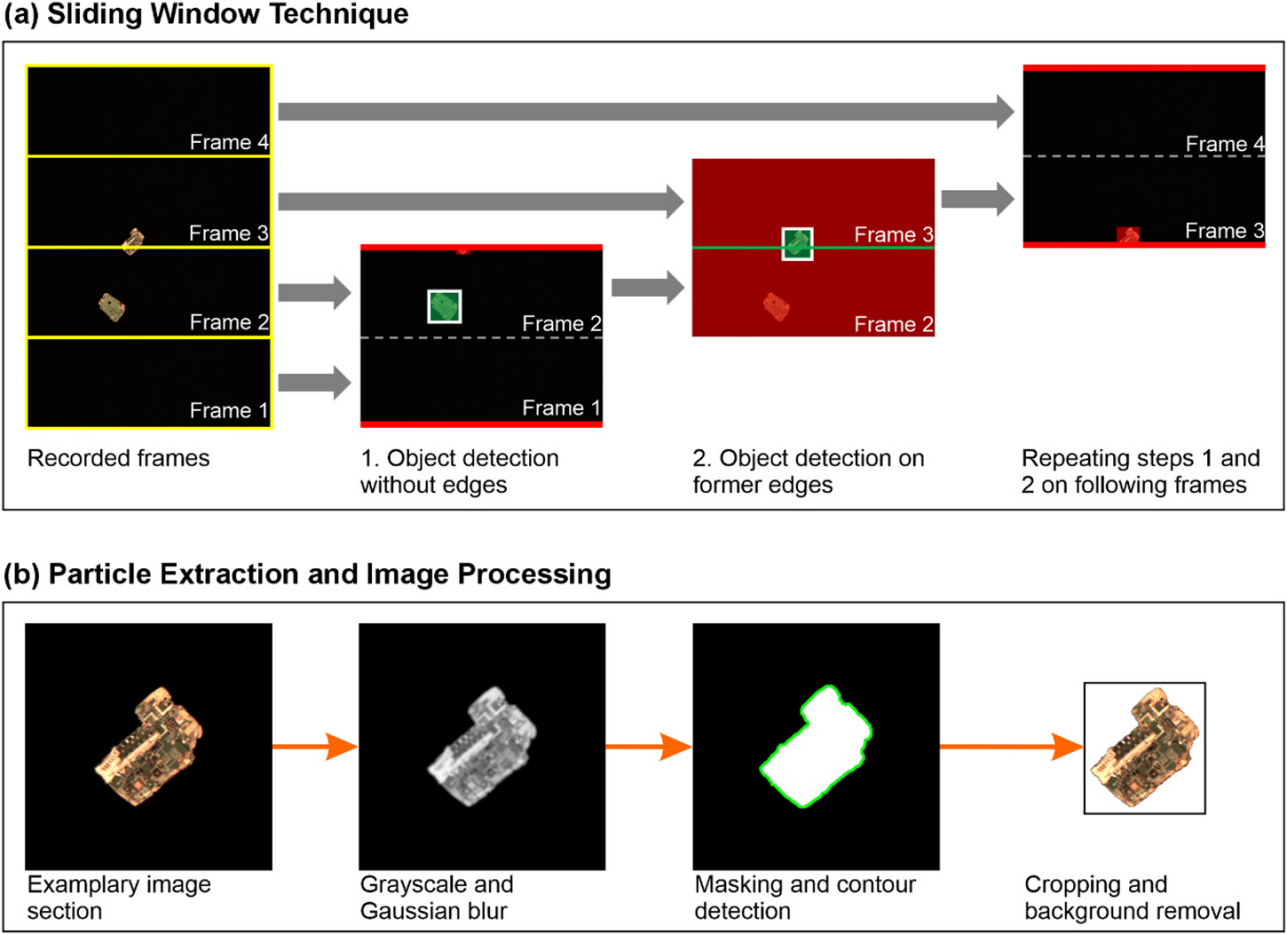
Principle of (a) the sliding window technique and (b) the particle extraction of complete objects from individual image frames (Adapted from [1]; cropped to the relevant part of image processing. © 2025 The Authors. CC BY 4.0).

**Sliding window technique:** Two sets of overlapping frame pairs were formed per camera (Fig. 7a): (1, 2), (3, 4), … and the offset pairs (2, 3), (4, 5), …. In the primary pairs, objects were detected if they did not touch the outer boundaries adjacent to the previous or next frames. For the offset pairs, only objects intersecting the central boundary between the two frames were detected. This ensures each object is captured exactly once in the subsequent extraction step.

**Particle extraction and image processing:** Particle masks were generated by converting to grayscale, smoothing with a 5- pixel Gaussian filter, and applying a fixed threshold of 10 on 8-bit (Fig. 7b). Contours were detected with OpenCV’s findContours, retaining the largest where overlaps occurred. Each particle was then cropped to the contour’s bounding box with 5- pixel padding on all sides.

#### 4.2.3 Weighing setup

To acquire paired particle images and masses for Dataset 2, the sorting function was disabled and single particles were manually fed onto the chute at approximately 4-s intervals. For each combination of material type and particle size range, ripple-divided portions of 100 particles (see Section 3.1) were processed sequentially, for up to 20 min or until the combination was exhausted. After imaging by the two RGB line-scan cameras, particles fell through a chute onto an output conveyor and were transported approximately 1 m before dropping into a container positioned on a digital precision balance (KERN & SOHN EWJ 3000–2 [Balingen, Germany]; 0.01 g resolution) (Fig. 6). Weight readings were logged every 125 milliseconds via USB with timestamps to enable one-to-one pairing with image data. For additional details on the conveyor and balance setup, see [1] and related work [9].

#### 3.2.4 Particle mass extraction

Particle images were matched to masses by aligning image timestamps with increases in the balance signal. Image timestamps were extracted from the cropped single-particle images and represent the moment each particle passed the RGB cameras. Following the approach in [9], these timestamps were used to identify the corresponding weight increases in the balance signal.

The transport time between the cameras and the balance was determined by calculating the delay between image timestamps and subsequent weight increases >1 g. The resulting distribution showed a mean and median of approximately 2.6 s, with a range of about 1.25 s – 4.0 s. The variability was attributed mainly to particles tumbling on the output conveyor immediately after landing. Based on this analysis, the automated mass extraction algorithm searched for weight increases within a window of 1.25 s – 5.0 s after the image acquisition time, but no later than 1.0 s after the next object’s image timestamp.

Because the impact of a single particle could register over several data points (due to measurement system inertia), the detection threshold was set to 0.8 g (below the 1 g analysis threshold). When an increase of ≥0.8 g was detected within the window, the particle mass was computed as the difference between pre- and post-event levels. To increase robustness to overshoot and lag, the median of the 1 s (8 samples at 125 ms) before the detected increase was subtracted from the median of the 1 s after it. Per-particle masses, together with image timestamps, were written to a CSV file. Timestamps without a detected increase in the search window remained unmatched (see match rate in Section 3.1.3).

To replicate the mass matching procedure, users can apply the settings provided above or: (1) estimate transport time by analyzing intervals between particle image timestamps and the next recorded clear weight increase (e.g., 0.5 g), (2) define an asymmetric search window based on median and spread, (3) add a minimum separation criterion to avoid overlaps of consecutive events based on minimum transport times, (4) set the threshold for step detection by analyzing weight increases per event, (5) use results of weight increase behavior to define window for pre- and post-event median, (6) calculate particle masses as the difference between pre- and post-event medians.

### 4.3 Data applications and performance benchmarks

This subsection illustrates how the datasets can be used to build and evaluate RGB-based SBMC pipelines. For full details see [1].

**Material type identification (DS1):** The single-particle images can be used to train computer vision models to identify ferrous metals (FEM), non-ferrous metals (NFM), printed circuit boards (PCB), and plastics (PLA) across two size classes. In [1], YOLOv11-based convolutional neural networks [10] were used for image classification, object detection, and instance segmentation, trained with 5-fold stratified cross-validation, and basic data augmentation (randomized flips/rotations). Object detection achieved mAP@0.5 ≈ 0.990 and mAP@[0.5:0.95] ≈ 0.990, instance segmentation reached mAP@0.5 ≈ 0.990 and mAP@[0.5:0.95] ≈ 0.986, and image classification reached top-1 accuracy ≈ 0.953.

**Particle mass prediction (DS2):** The paired single-particle images and masses can be used to train regression models to predict particle masses from 2D images with material type information. In [1], features were extracted with OpenCV [7], scikit-image [8] and imea [11] to generate 53 shape/size features and 13 color features (HSV statistics and specularity), combined with one-hot encoded material type. Models were implemented in scikit-learn [12] (e.g., linear, Lasso, support vector, random forest, gradient boosting, k-nearest neighbors (k-NN), neural network) with 5-fold cross-validation and min-max scaling. Evaluation used MCRE (mean of the absolute class-wise relative errors between predicted and true total masses) and RTE (relative error of the predicted total mass). Best results were achieved with k-NN regression, reaching mean MCRE ≈ 4.89 % across folds and mean RTE ≈ 2.00 %.

**MFCO validation (DS3):** The single-particle images and ground-truth material masses per mixed sample can be used to validate predictions of material flow compositions. In [1], MTI (DS1) and PMP (DS2) were combined and applied to DS3. Two variants were tested: (1) object detection on single-particle images with k-NN regression on extracted features; (2) instance segmentation on synthetic collages with k-NN regression on features from predicted masks. Predicted particle masses were aggregated by class and compared with ground-truth compositions of the three mixed samples. Variant (1) achieved mean MCRE ≈ 4.94 % across mixtures and RTE between −5.58 % and 1.17 % per mixture. For the combined sample (MS1 – MS3), it achieved MCRE ≈ 4.63 % and RTE ≈ −2.44 %. Variant (2) showed higher errors (mean MCRE ≈ 10.8 %).

## Limitations

The datasets exclude particles smaller than 12.5 mm and larger than 50 mm, which were removed during sieving. Black plastics were omitted to improve detectability against the black conveyor belt of the sensor-based sorting machine. Metals with colored coatings were excluded to increase optical contrast between metals and plastics. Materials outside the four investigated classes (ferrous metals, non-ferrous metals, PCBs, plastics), as well as mixed-material objects, were not included. These exclusion criteria result in Dataset 1 covering approximately 75 % of the mass of the investigated streams within the two size ranges. Additionally, not all fractions produced at the WEEE treatment plant were analyzed, i.e., fractions consisting solely of < 12.5 mm particles (e.g., fine copper from cable shredder material and fine plastic residue) were excluded. Produced quantities for each fraction and material at the WEEE plant were not available.

These design choices improve ground-truth quality and comparability but reduce representativeness of the full WEEE stream and introduce a visibility bias. Reported MTI and MFCO performance may overestimate results on real streams containing dark plastics, coated metals, wires, cables, mixed-material particles and classes not included. The samples were collected during a single campaign at a WEEE plant. Composition, coatings, illumination, sensors, and image background may vary by site and over time. For broader deployment and future research, users should consider extending the data with site-specific samples (including dark plastics and coated metals), and validation under local lighting and sensor conditions.

Because recording individual particles is time-consuming, Dataset 2 contains only a subset of the particles from Dataset 1. Manual feeding and the collection on a precision balance can introduce pairing errors that lead to unmatched images. If the interval between successive particles is too short, their events are excluded by the extraction algorithm. Particles that bounce or roll on the output conveyor may miss the collection box or create overlapping weight steps with subsequent particles, also leading to exclusion. Another potential source of error, which was not observed but cannot be ruled out, is the simultaneous feeding of several (e.g., stuck) particles. This can cause overlap, which may distort geometric features and recorded masses. Consequently, the matching of mass and image data in Dataset 2 may contain a small number of errors.

Overlap is more likely during bulk feeding. In Dataset 1, its impact is expected to be low due to mono-material streams and a focus on MTI, which likely relies on features that are relatively evenly distributed across particles. In Dataset 3, overlap may produce images containing multiple material types, distorted geometric features, or missed detections when particles are fully occluded.

## Ethics Statement

The authors declare to have read and follow the ethical requirements for publication in Data in Brief and confirm that the current work does not involve human subjects, animal experiments, or any data collected from social media platforms.

## CRediT Author Statement

**Malte Vogelgesang**: Conceptualization, Methodology, Software, Validation, Formal Analysis, Investigation, Data Curation, Writing – Original Draft, Visualization. **Alice do Carmo Precci Lopes**: Writing – Review & Editing. **Emanuel Ionescu**: Writing – Review & Editing, Supervision. **Liselotte Schebek**: Writing – Review & Editing, Supervision.

## Data Availability

TUdatalibDataset for automated material flow characterization of shredded WEEE: RGB-camera-based object images and mass data (Original data).
